# Meal Replacement Beverage Twice a Day in Overweight and Obese Adults (MDRC2012-001)

**DOI:** 10.2174/157340112803832156

**Published:** 2012-11

**Authors:** Joy L Frestedt, Lindsay R Young, Margie Bell

**Affiliations:** 1 Alimentex^TM^, Minnesota Diet Research Center, Frestedt Incorporated, St Louis Park, MN,; 2 ClinData Services, Inc., Fort Collins, CO, USA

**Keywords:** Hunger, meal replacement beverage, obesity, satiety, SF-36, SLIM questionnaire, therapeutic lifestyle changes diet, three factor eating questionnaire, weight loss.

## Abstract

This open label, single arm, prospective, interventional, weight loss trial evaluated a meal replacement beverage (Right Size^®^ Smoothie) used to replace breakfast and lunch each day for 12 weeks (7 clinic visits) as part of a calorie-restricted diet in overweight and obese adults. A total of 155 individuals were screened, 55 enrolled and 28 completed this 12 week study. Subjects were obese (mean weight: 206 pounds and BMI: 32.7 kg/m^2^) and the mean age was 40 years including 42 (76.4%) female and 13 (23.6%) male volunteers. The modified Intent to Treat and Completer groups lost an average of 10.6 and 13.8 pounds and reduced their average BMI by 1.7 and 2.2 kg/m^2^ respectively during this 12 week trial. The Per Protocol group lost 15.2 pounds and 2.4 kg/m^2^ and the Optimal Weight Loss group lost 18.5 pounds and 2.9 kg/m^2^. Using the Satiety Labeled Intensity Magnitude scale (SLIM) questionnaire, subjects reported feeling relatively hungry before they consumed the beverage, then feeling relatively full 15 minutes following the beverage with the sensation of some fullness lasting more than 2 hours and then feeling relatively hungry again at 3 hours after consuming the beverage. Study subjects reported significant improvements in physical functioning, general health, vitality and mental health as well as increased cognitive restraint of eating, reduced disinhibition and reduced hunger during the trial. The study beverages were well tolerated and no Serious Adverse Events (SAE) reported. This study suggests the study beverage aids in weight loss by helping to curb hunger during a reduced calorie diet program.

## INTRODUCTION 

Obesity is a chronic problem in North America with serious implications for health, disease states, and, ultimately, loss of life. Health problems linked to obesity include diabetes mellitus, cardiovascular disease, osteoarthritis, and certain forms of cancer. This obesity epidemic continues to worsen despite the compelling evidence that obesity is linked to premature death from all causes [1-3]. Some authors suggest obesity may be replacing under-nutrition and infectious disease as the main causes of ill health [4, 5]. Treatment of obesity has become a major focus for improving health in industrialized countries and the World Health Organization (WHO) [6] has proposed specific definitions for obesity based on the Body Mass Index (BMI) (BMI=Body Weight (kg) / [Body Height (m)]2). Catagories of BMI include underweight (<18.5 kg/m^2^), normal weight (18.5-24.9 kg/m^2^), overweight (25-29.9 kg/m^2^), obese (30-39.9 kg/m^2^) and morbidly obese (≥ 40.0 kg/m^2^).

The Center for Disease Control and Prevention indicates more than 72 million people and 17% of children in the US are obese with obesity rates doubling for adults and tripling for children in the 28 years between 1980 and 2008 [7]. Obesity is a problem for all population groups (including all ages and education levels) and more than ever before Americans are attempting to diet and lose weight in order to improve their overall health. Unfortunately, for most individuals who are overweight or obese, the goal of getting to an ideal weight or body fat percentage may be practically unachievable since most diets end in failure. Fortunately, even small reductions in weight (5-10%) can substantially improve blood pressure, serum lipid levels, and glucose tolerance and diminish the incidence of diabetes and hypertension [7-10] although greater weight loss may be necessary for long-term risk reduction [11].

Weight loss requires a period of negative energy balance that tends to be difficult to reach and maintain in individuals who are susceptible to weight gain. Liquid meal replacement beverages have been shown to aid in weight loss in combination with an energy-restricted diet [12, 13] and during weight loss maintenance [13, 14]. The success of meal replacement beverages may be due to the portion-controlled nature of a meal replacement, the ease of use (i.e. reduction in meal-planning and preparation) and reduction in the temptation of choosing high calorie foods for the meals replaced. A meta-analysis that analyzed 6 studies of liquid meal replacements indicated that weight loss was greater in the meal replacement groups compared to the reduced calorie traditional diets while the calorie goal for the groups was equivalent [15].

The meal replacement beverage studied in this clinical trial was specially formulated for this trial to have a decadent flavor, texture, and mouth feel and contains protein, fiber and crystalline fructose along with more than 20 vitamins and minerals. Prior research suggests protein and fiber play important roles in satiety and weight management [16-19]. Additionally, fructose (a low glycemic index carbohydrate) [20] may also aid in satiety and weight loss since insulin is not required for the first step of fructose digestion (in the liver) [21]. Fructose appears to suppress food intake while affecting plasma insulin levels [22] and preventing a post-prandial rise in blood glucose [23, 24]. The primary objective of this open label, single arm, prospective, interventional trial was to evaluate the effect of the study beverage on weight loss in overweight and obese adults when the beverage was used to replace breakfast and lunch every day for 12 weeks in the setting of a calorie-restricted at-home diet. Feelings of satiety and fullness were also assessed during this trial.

## MATERIALS AND METHODS

### Ethics

This study was performed in compliance with all applicable laws, regulations and guidelines (e.g. the Belmont Report, Declaration of Helsinski, 21CFR50-Protection of Human Subjects, and 21CRF56-Institutional Review Boards). Schulman Associates Institutional Review Board (SAIRB, Cincinnati, OH) provided oversight for this trial and approved the protocol, informed consent document, subject recruitment materials and relevant supporting information prior to the initiation, during the conduct and after completion of the study. Subjects volunteered and provided written, informed consent before the start of any trial-related activities. 

### Study Volunteers

Volunteers were recruited for this study using flyers posted in nearby businesses, emails to near-by clinic staff, and postings to on-line forums. Study volunteers were included if they were generally healthy, 20-55 years old and had a BMI between 26 and 40 kg/m^2^. Study volunteers were excluded if they were pregnant, had digestive, absorptive or gall bladder impairments, or a history of osteoporosis, diabetes (Type 1 or Type 2 diabetes mellitus), uncontrolled hypertension, kidney disease, thyroid disease, hematological disease, unstable cardiovascular disease, eating disorder, hepatic, pulmonary or renal disease, active cancer, HIV infection, or surgery for weight loss. Volunteers were also excluded if they started or quit smoking in the past 3 months, lost (or gained) more than 10 pounds in the past 3 months, exercised regularly (i.e. more than 30 minutes per day), had evidence of alcohol or drug abuse in the past 2 years, had taken medications or supplements for weight loss in the past 14 days or needed diuretics or thyroid replacement treatments.

### Study Beverage

The study beverage Right^®^ Size Smoothie (Insight Beverages^®^, Inc., Lake Zurich, IL, **Appendix A**) was dispensed as a powdered drink mix and each subject was given sufficient beverages for 20 days of consumption (40 beverage mixes) even though each subject was asked to return all unopened beverage in two weeks (14 days). Subjects were instructed to mix the study beverage (29g) with 8 ounces of skim milk, water or other liquid (e.g. soy milk, coconut milk, etc.) and to consume the entire study beverage (followed by 8 ounces of water) to replace breakfast and lunch each day for 12 weeks.

### Diet Program

The diet program was similar to prior work [25] and the standard “balancing calories” paradigm set by the Centers for Disease Control and Prevention [26]. Each subject was placed on an appropriate calorie-restricted diet during their individual diet counseling session using the formula: Basal Metabolic Rate (BMR) [27] x 1.3 activity factor – 500 kcal. This calorie restriction of 500 calories/day (i.e. subtracting 500 kcal from the Total Energy Expenditure predicted by the Harris Benedict Equation) was expected to create a negative energy balance designed to result in a weight loss of 1 pound per week. A range of diet instruction sheets (including 1000, 1200, 1400, 1600, 1800, 2000, 2200, and 2400 calorie diets) were used to help subjects comply with their specific calorie-restricted diet and subjects were provided dietary counseling and reading materials to help them comply with calorie counting [28] and managing their diet.

### Study Visits

At the baseline visit, subjects who fit the enrollment criteria were given study information, signed an informed consent form, discussed their medical and medication history and completed the 24-hour dietary recall as well as three questionnaires: 1) Rand version 1 SF-36 [29], which provides a profile of functional health and well being, physical and mental health along with a health index measuring eight areas including physical functioning, bodily pain, role limitations due to physical health problems, role limitations due to personal or emotional problems, emotional well-being, social functioning, energy/fatigue and general health perceprions, 2) Three Factor Eating Questionnaire (TFEQ) [30], which measures three dimensions of human eating behavior: cognitive restraint of eating, disinhibition and hunger; and 3) Satiety Labeled Intensity Magnitude (SLIM) Scale questionnaire [31], which measured on a 100 point scale ranging from -100 indicating greatest imaginable hunger to +100 indicating greatest imaginable fullness with 0 in the middle of the scale indicating neither hungry nor full. Clinic staff measured their height and weight and prescribed their calorie-restricted diet (approximately 500 calories/day less than the total energy expenditure based on baseline Harris Benedict equation and review of 24 hour food recall). Subjects were given the study beverage, a diet diary, beverage consumption log and SLIM questionnaires and they were instructed to record their food intake, time of beverage consumption and feelings of fullness on each of three (3) days during the next two weeks. Subjects were scheduled to return to the clinic every two weeks for a total of 12 weeks. For all visits after the baseline visit, subjects were weighed and they returned their unopened product, beverage consumption log, diet diary and questionnaires. Clinic staff reviewed and discussed the diet diaries, questionnaires, any changes in medical history, medications, adverse events or product complaints. Subject compliance with the study protocol and all study procedures was assessed at each visit by test article count, review of diet diaries and discussion with the subject. At the final visit (after 12 weeks of treatment), subjects were weighed and staff discussed their final weight.

### Statistical Analyses

Target enrollment was determined by review of similar weight loss studies [16, 17, 25] and a target enrollment of a minimum of 50 subjects was determined to end up with 25 subjects completing the trial (allowing for a 50% attrition rate). Study data and records were stored in the secured study center and records identifying the study subject were kept confidential. Data was periodically entered into a database and checked for accuracy. An independent statistician provided descriptive statistics and standard statistical techniques to determine if significant weight loss occurred. A modified intent to treat (mITT) population was evaluated and a last observation was carried forward (LOCF) and imputed for any missing data (i.e. the data from the prior visit/observation was used to replace any missing data at a subsequent visit). The subjects only present for the baseline visit were not included in the mITT population since they did not have a second value (i.e. no data were available from a second visit after they had started to consume the product) to carry forward. A Completer population included all subjects who completed the trial. A Per Protocol (PP) population included all subjects who completed the trial by generally following the instructions provided during the trial as described in the protocol and an Optimal Weight Loss (OWL) population included only those subjects who lost more than 0.7 pounds each week on average and did not gain more than 0.3 pounds during any two week period during the trial. For the satiety measures over time, an Area Under the Curve (AUC) value was calculated using the “incremental AUC” method where any values below zero were excluded from the calculation. Baseline (BL) and end of study (EOS) data were analyzed with paired t-tests using SAS statistical software (SAS Institute, Cary, NC). A p-value of 0.05 was considered statistically significant. Data values are presented as mean ± standard error of the mean (SEM).

## RESULTS

**Fig. (1):**
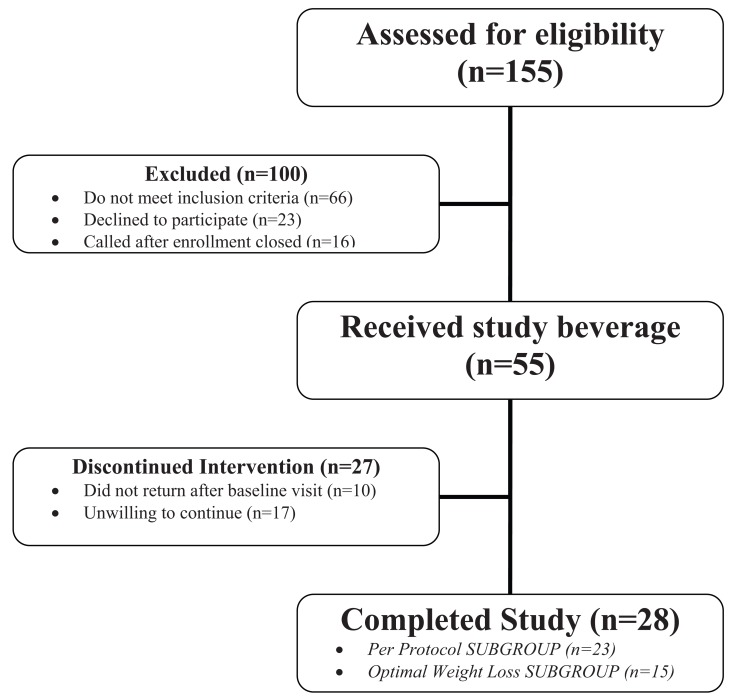
Flow diagram of progress through the trial.

A total of 155 individuals were screened for this study. Most of the 100 people excluded during screening had a BMI either higher or lower than our BMI range of 26-40 kg/m2. Many others were excluded because they were too old or had an excluded medical condition or medications. Those that declined to participate either were not interested in the study after hearing the details or did not show for their enrollment visit and were non-responsive to calls to follow-up. Fifty-five (55) healthy adults gave voluntary, written informed consent and were enrolled into the study and 28 individuals completed the study (Fig. **1**). The “mITT” (modified Intent to Treat) group included 45 subjects who provided written informed consent and returned for at least one visit after the baseline. The “Completer” group included 28 subjects who completed the trial and the “Per Protocol” group included the 23 subjects who completed the trial according to the protocol (no missed visits and returning documents as required, etc). The “Optimal Weight Loss” group included all 15 subjects who were the most compliant with weight loss activities during the trial. Twenty-seven (27) subjects discontinued participation for the following reasons: 10 did not show up for any more visits after the baseline visit or BMI was too high at baseline, 8 were unwilling to continue, to complete paperwork or to follow the diet, 5 had scheduling issues, job/travel, family obligations, or emergency/death in family, 1 stopped taking product, 1 was bored with trial, 1 had nausea and 1 had diarrhea and did not wish to continue.

### Baseline Demographics

Subjects were predominantly obese women and slight differences in demographics were noted between the sub-groups (Table **1**). As expected, the average male diet plan was slightly more than 1700 kcal while the average female diet plan was slightly more than 1100 kcal.

### Changes in Body Weight and Body Mass Index

**Fig. (2):**
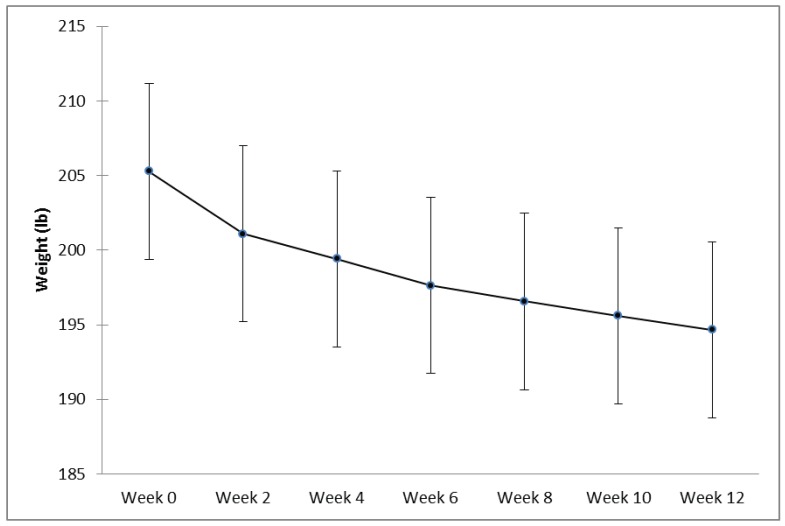
Weight (in pounds) over time from Week 0 (baseline) to Week 12 (end of study) for the modified Intent To Treat (mITT) group (n=45) (mean +/- SEM).

Subjects in the mITT (n=45) and Completer (n=28) groups lost an average of 10.60 ± 1.23 pounds (p<0.001) (Fig. **2**) and 13.81 ± 1.45 pounds (p<0.001) and reduced their average BMI by 1.68 ± 0.19 and 2.22 ± 0.22 kg/m^2^ respectively during this 12 week trial. The Per Protocol group lost an average of 15.17 ± 1.56 pounds (p<0.001) and 2.43 ± 0.23 kg/m^2^ and the Optimal Weight Loss group lost 18.46 ± 1.74 pounds (p<0.001) and 2.94 ± 0.24 kg/m^2^. Of interest, within the mITT population, five subjects went from a baseline BMI at week 0 indicating “obese” to a BMI indicating “overweight” and two subjects moved from a BMI indicating “overweight” to a BMI indicating “normal” at week 12.

### Feelings of Fullness and Satiety

**Fig. (3):**
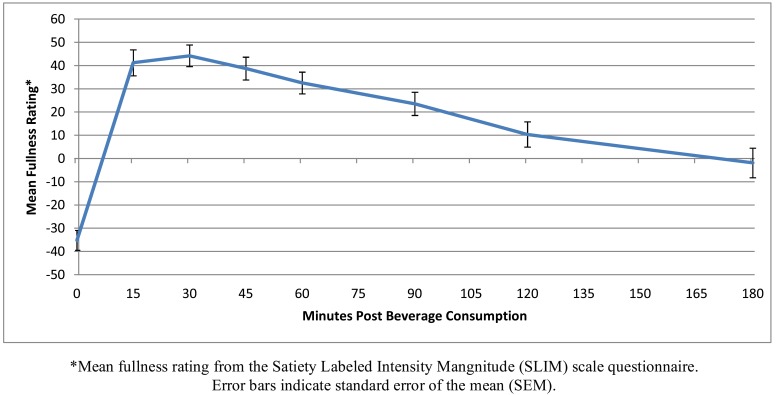
Satiety Labeled Intensity Magnitude (SLIM) scores modified Intent to Treat (mITT) population.

Subjects documented feeling full for more than 2 hours following consumption of the study beverage: 2.49 ± 0.13 hours for the mITT group, 2.44 ± 0.14 hours for the Completers group, 2.38 ± 0.15 hours for the Per Protocol group and 2.35 ± 0.19 hours for the Optimal Weight Loss group. SLIM scores generally changed from relatively hungry before consuming the beverage (range of means: -35.22 to -40.65 among groups on a 100 point scale) to relatively full starting at 15 minutes following the beverage (range of means: 37.13 to 41.46) and lasting 2 hours (range of means at 120 minutes: 4.96 to 10.34) and then feeling relatively hungry again at 3 hours (range of means: -1.90 to -10.00) after consuming the beverage. A representative curve showing these changes over time is presented for the mITT population (Fig. **3**).

### Changes in Health Survey Measures (SF-36)

**Fig. (4):**
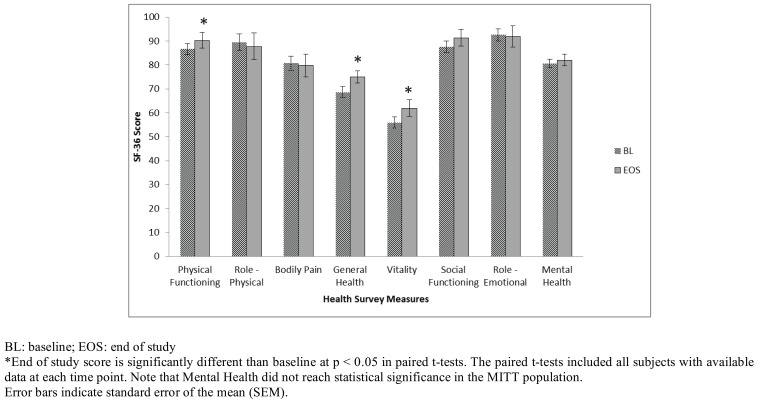
Changes in Health Survey Measures for the modified Intent To Treat (mITT) population.

The RAND 36-Item Health Survey (Version 1.0) is identical to the Medical Outcomes Study (MOS) SF-36 and measures physical functioning, bodily pain, role limitations due to physical health problems, role limitations due to personal or emotional problems, emotional well-being, social functioning, energy/fatigue and general health perceptions. All scores for the completers, per protocol and optimal weight loss groups increased over the 12 weeks in this trial and within these subgroups the increases were significant for physical functioning, general health, vitality and mental health. The scores for the mITT group are depicted in Fig. **4**.

### Changes in Three Factor Eating Questionnaire (TFEQ)

**Fig. (5):**
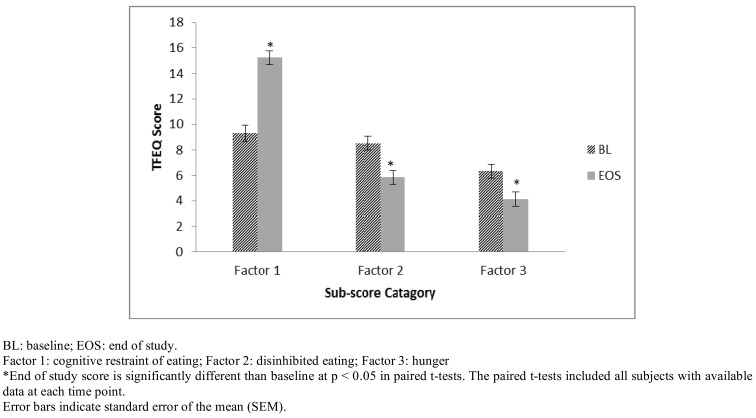
Changes in Three Factor Eating Questionnaire (TFEQ) scores for the modified Intent to Treat (mITT) group.

Three Factor Eating Questionnaire scores were significantly increased for Factor 1 (cognitive restraint of eating) while scores were significantly decreased for Factor 2 (disinhibited eating) and Factor 3 (hunger) from Baseline to End of Study regardless of the subgroup analyzed. The scores for the mITT group are depicted in Fig. **5**. 

### Adverse Events

The study beverages were well tolerated and no Serious Adverse Events (SAE) were reported. A total of 25 Adverse Events (AE) were documented in 20 study subjects which were not believed to be related to the study beverage including 10 reports of upper respiratory infection or flu, 3 reports of nausea or stomach ache, 2 reports of migraine and 1 report each of Bell’s Palsy, constipation, diarrhea, food poisoning, musculoskeletal pain secondary to motor vehicle accident, surgery for bone spurs in right shoulder, tachycardia secondary to lead malfunction in pacemaker, urinary tract infection, wisdom tooth removal secondary to headaches, and worsening hip/back pain.

## DISCUSSION

This open label, prospective clinical trial evaluated the effect of the trial beverage on weight loss when used to replace breakfast and lunch each day in the setting of a calorie-restricted at-home diet. Twenty-eight (28) subjects completed the trial which was slightly more than the 25 expected according to the protocol and based on the results of previous research which was used to predict the optimal sample size at the end of the study [16, 17, 25]. As expected, subject compliance with the diet was variable throughout the study; however significant weight loss did occur during the 12 weeks of the trial. The selected sub-groups of subjects lost an average of 10.6, 13.8, 15.2 and 18.5 pounds over the 12 weeks in the trial (mITT, Completer, Per Protocol Optimal Weight Loss subgroups, respectively). These results were expected since the calorie-restricted diet was designed to decrease body weight by about 1 pound a week or 12 pounds over the 12 weeks of this trial.

Of particular interest, roughly 10% of participants moved from a baseline BMI at week 0 indicating “obese” to a BMI indicating “overweight” and an additional ~5% moved from a BMI indicating “overweight” to a BMI indicating “normal” weight at week 12. This finding suggests significant health improvement for those study subjects who moved to a less obese group according to the World Health Organization obesity definitions. For example, the participants who completed the study lost an average 4.8% of their total body weight which is another way to measure this significant health benefit since even small reductions in weight (like 5-10%) can substantially improve blood pressure, serum lipid levels, and glucose tolerance and may diminish the incidence of diabetes and hypertension [7-10]. A future clinical trial may be helpful to evaluate the duration of weight loss since a particular concern is that subjects may gain back the weight they lost if they have not successfully changed their lifestyle and food choices.

This study measured changes in satiety over time after consuming the meal replacement beverage and subjects indicated they moved from feeling relatively hungry before consuming the beverage to feeling relatively full 15 minutes following the beverage. This feeling of fullness lasted more than 2 hours and SLIM scores indicated they were feeling relatively hungry again at 3 hours after consuming the beverages. The Area Under the Curve (AUC) for the SLIM scores were calculated to measure the amount of time when a subject was not hungry. When using this instrument, we noticed the AUC values were significantly higher at the end of the study than at the beginning of the study; however, this was attributed (at least in part) to the training needed for subjects to learn how to score their hunger and satiety using the SLIM tool. Additional research may be helpful to see if time on the study beverage actually improves satiety during weight loss as measured using this SLIM tool.

The Health Survey Questionnaire showed significant improvements in physical functioning, general health, and vitality (and mental health in the sub-populations studied) sub-scores from baseline to the end of the trial. The physical functioning scale is defined as measuring the ability to perform physical activities from bathing or dressing to the most vigorous activities without limitations due to health. This improvement in physical functioning suggests a benefit associated with the weight loss which might be expected when an overweight/obese person loses ~5% or more of their body weight. The general health scale is defined as measuring the individual’s evaluation of their personal health from poor (and likely to get worse) to excellent. This improvement in general health suggests a change in the way the study subject’s perceived their overall health and indicates they evaluated their personal health as improving during the 12 weeks in the trial. The Vitality scale is defined as measuring the feeling of being tired and worn out all the time to feeling full of pep and energy all of the time. This improvement in vitality suggests study subjects felt they have more energy/pep at the end of the trial which might be associated with having less weight to move around. The mental health scale is defined as measuring feelings of nervousness and depression al the time to feelings of peace, happiness and calm all the time. This improvement in mental health suggests study subjects may have benefited emotionally from their success in the weight loss program. Compared to the general US adult population, the completer population in this study scored above the national average for all scales except general health and vitality at baseline (Table **2**). This expected finding for obese/overweight persons compared to the US national averages warrants additional research since study participants scores improved and all scores were well above the national average at the end of the study. Additional research may be warranted to ensure these findings are directly related to weight loss and the extent to which the study beverage may have contributed to these health benefits.

The Three Factor Eating Questionnaire (TFEQ) measures three dimensions of human eating behavior: cognitive restraint of eating, disinhibition and hunger. Higher scores indicate restrained eating, disinhibited eating and predisposition to hunger, respectively. Scores in the TFEQ were significantly increased from baseline to end of study for Factor 1 (increased cognitive restraint of eating) while scores were significantly decreased for Factors 2 (reduced disinhibition) and 3 (reduced hunger). These results suggest the study subjects increased their ability to modify their eating habits and “restrain” their eating intentionally, for example, by avoiding fattening and high calorie foods. This modification may be based (in part) on the calorie knowledge they learned during the trial and on the ease of using the study beverage to intentionally manage calories consumed at breakfast and lunch. Subjects had a measurable increase in conscious, self-directed behavior to improve health [30]. Study subjects were also able to decrease their susceptibility to eating problems (disinhibited eating) and hunger. Part of the habitual susceptibility to eating problems may be related to the cyclical nature of restraint versus disinhibited eating and subjects may have benefited because the study beverages replaced meals and provided them an easy way to control at least part of their eating problem behavior as they became more cognitively aware of their disinhibited eating habits and learned to control those behaviors. For example, during this trial, subjects may have become more aware of their internal and external cues for hunger as they reduced their food consumption. Additional research may be helpful to determine if subjects who fail to lose weight or who drop out from a trial may have more severe poor eating behaviors.

The weight loss observed in this trial is comparable to that of other trials of equal or greater duration involving meal replacement beverages [15, 33, 34]. In the meta-analysis performed on trials conducted in 2001 or earlier [15], significant weight loss (approximately 7% of baseline body weight) overall was obtained in the six studies at 12 weeks and weight loss was significantly greater than in a reduced calorie diet alone in the meta and pooled analyses. Meal replacement beverages have also been shown to aid in weight maintenance or further weight loss in subjects that lost at least 5% of their body weight following weight loss [14]. The results of one study [33] indicated subjects randomized to the meal replacement arm of the trial had greater adequacy of micronutrient intake during weight loss than the traditional food group. Although no biochemical measurements were made during this trial, other studies have shown that symptoms of the metabolic syndrome [35, 36] and oxidative stress and inflammatory biomarkers (C-reactive protein and urine lipid peroxides, respectively) [37] were reduced with weight loss using meal replacement beverages. Future studies may include biochemical and body composition measurements to identify changes in risk factors with weight loss using the test beverage in comparison with other treatments. Few meal replacement studies incorporated satiety measures or quality of life measures. One study [37] assessed satiety at 0 and 16 weeks and found no difference in satiety between the meal replacement group and the food-based group.

A purpose of this trial, in addition to weight loss and satiety measures, was to capture subject satisfaction related to the study beverage. The beverage was reformulated to improve the taste profile prior to conduct of the trial and testing the beverage in a clinical setting was the first step to substantiating the effects of the beverage to be satisfying enough for the participants to adhere to the reduced calorie diet and lose weight. 

The study beverage and study diet were well tolerated and no serious adverse events were reported. Mild gastrointestinal discomfort or disturbances (e.g. abdominal rumbling, gas/bloating, soft stools or limited constipation) were expected as is common with individuals who change their dietary consumption to increase the relative amounts of fruits and vegetables in their diets and to reduce high calorie foods. Only five subjects reported any symptoms of nausea, stomach ache, diarrhea or constipation and these were all mild and self-limiting. Two individuals elected to discontinue the trial due to diarrhea in one case and nausea in the other case. These adverse events were difficult to assign specific, unequivocal relationship to the study beverage due to concurrent illnesses being treated: the participant with diarrhea also had an upper respiratory tract infection at the time of the adverse event and the participant with nausea was also taking antibiotics for the treatment of a urinary tract infection at the time of the adverse event. 

## CONCLUSIONS

The study beverage was well tolerated and subjects who completed the trial lost an average of 13.8 pounds over 12 weeks. This study suggests the RightSize^®^ Smoothie supported feelings of fullness for more than 2 hours on average and the weight loss program produced significant improvements in physical functioning, general health, vitality, mental health, cognitive restraint of eating, reduced disinhibition and reduced hunger. This study suggests RightSize^®^ Smoothie aids in weight loss when used to replace both breakfast and lunch meals each day for 12 weeks in the setting of a calorie-restricted at-home diet. In addition, this trial helps to advance the science of meal replacement beverages for weight loss by allowing the development of future clinical trials which might now be powered to show significant differences between the Right Size^®^ Smoothie beverage and an appropriate comparator treatment.

## Figures and Tables

**Table 1.** Baseline Demographics

Characteristic	ALL (n=55)	SEM	mITT (n=45)	SEM	Completer (n=28)	SEM	PP (n=23)	SEM	OWL (n=15)	SEM
Sex (female)	42 (76.4%)	-	35 (77.8%)	-	24 (85.7%)	-	19 (82.6%)	-	12 (80.0%)	-
Age (years)	40.05	1.41	39.77	1.57	39.60	1.99	37.80	2.20	35.09	2.35
Height (in)	66.45	0.51	66.43	0.54	65.61	0.55	65.83	0.62	65.89	0.82
Weight (lb)	206.04	5.35	205.28	6.05	197.54	6.76	198.29	7.98	201.49	10.34
BMI (kg/m2)	32.66	0.57	32.52	0.63	32.12	0.75	31.99	0.86	32.45	1.14
BMR: Male	2275.1	75.93	2316.9	79.33	2269.9	128.61	2269.9	128.61	2246.5	178.87
Diet plan: Male	1775.1	75.93	1816.9	79.33	1769.9	128.61	1769.9	128.61	1746.5	178.87
BMR: Female	1616.3	22.20	1610.4	24.78	1602.3	26.66	1605.8	32.28	1633.5	43.18
Diet plan: Female	1116.3	22.20	1110.4	24.78	1102.3	26.66	1105.8	32.28	1133.5	43.18

mITT=modified Intent To Treat; Completer=completed the trial; PP=Per Protocol; OWL=Optimal Weight Loss, SEM=Standard Error of the Mean

**Table 2.** Comparison of the Means for Each of the Eight Scales Between the Completer Population (at Baseline and End of Study) and the General U.S. Adult Population [29]

SF-36 Scale	US	Baseline	End of Study
Physical Functioning	84.2	86.61	92.32+
Role - Physical	80.9	84.82	100.89
Bodily Pain	75.2	78.48	82.41
General Health	71.9	68.57-	77.50+
Vitality	60.9	51.79-	63.39+
Social Functioning	83.3	87.95	94.20
Role - Emotional	81.3	90.48	97.62
Mental Health	74.7	78.57	83.43+

- Scores below the US average

+ Significant improvement from Baseline to End Of Study

## APPENDIX A: TEST ARTICLE NUTRITION INFORMATION

### Lean Cocoa Bean Smoothie Mix – EXB3350134

**INGREDIENTS:** SOY PROTEIN ISOLATE, FRUCTOSE, RESISTANT MALTODEXTRIN (SOLUBLE DIETARY FIBER), COCOA (PROCESSED WITH ALKALI), CANOLA OIL, **VITAMIN AND MINERAL BLEND** [DICALCIUM PHOSPHATE, DIMAGNESIUM PHOSPHATE, ASCORBIC ACID, VITAMIN E ACETATE, NIACINAMIDE, ZINC CITRATE, D-CALCIUM PANTOTHENATE, VITAMIN A PALMITATE, PYRIDOXINE HYDROCHLORIDE, MANGANESE SULFATE, RIBOFLAVIN, THIAMIN MONONITRATE, COPPER GLUCONATE, FOLIC ACID, BIOTIN, CHROMIUM PICOLINATE, POTASSIUM IODIDE, PHYTONADIONE, SODIUM MOLYBDATE, SODIUM SELENITE, CHOLECALCIFEROL, CYANOCOBALAMIN (VITAMIN B12)], CORN SYRUP SOLIDS, NATURAL AND ARTIFICIAL FLAVORS, SILICON DIOXIDE (AN ANTICAKING AGENT), CARBOXYMETHYLCELLULOSE GUM, XANTHAN GUM, SALT, SODIUM CASEINATE (A MILK DERVIATIVE) GREEN TEA EXTRACT, DIPOTASSIUM PHOSPHATE, ACESULFAME POTASSIUM, MONO- AND DIGLYCERIDES, SOY LECITHIN, GINGER ROOT EXTRACT, SUCRALOSE, CINNAMON BARK EXTRACT.

**Contains Milk, Soy.**

### Skinni Vanilli Smoothie Mix EXB3350136

**INGREDIENTS:** SOY PROTEIN ISOLATE, RESISTANT MALTODEXTRIN (SOLUBLE DIETARY FIBER), FRUCTOSE, CANOLA OIL, **VITAMIN AND MINERAL BLEND** [DICALCIUM PHOSPHATE, DIMAGNESIUM PHOSPHATE, ASCORBIC ACID, VITAMIN E ACETATE, NIACINAMIDE, ZINC CITRATE, D-CALCIUM PANTOTHENATE, VITAMIN A PALMITATE, PYRIDOXINE HYDROCHLORIDE, MANGANESE SULFATE, RIBOFLAVIN, THIAMIN MONONITRATE, COPPER GLUCONATE, FOLIC ACID, BIOTIN, CHROMIUM PICOLINATE, POTASSIUM IODIDE, PHYTONADIONE, SODIUM MOLYBDATE, SODIUM SELENITE, CHOLECALCIFEROL, CYANOCOBALAMIN (VITAMIN B12)], CORN SYRUP SOLIDS, MALTODEXTRIN, NATURAL AND ARTIFICIAL FLAVORS, SILICON DIOXIDE (AN ANTICAKING AGENT), XANTHAN GUM, CARBOXYMETHYLCELLULOSE GUM, SALT, SODIUM CASEINATE (A MILK DERIVATIVE) GREEN TEA EXTRACT, DIPOTASSIUM PHOSHPATE, ACESULFAME POTASSIUM, MONO- AND DIGLYCERIDES, SOY LECITHIN, GINGER ROOT EXTRACT, SUCRALOSE, CINNAMON BARK EXTRACT.

**Contains Milk, Soy.**
